# The economic case for increased investment in the treatment of adolescents with common mental disorders in Colombia and South Africa

**DOI:** 10.1093/heapol/czag075

**Published:** 2026-09-30

**Authors:** Kim Sweeny, Nicola Reavley, Bruce Rasmussen, Hlamalani Queen Nenzhelele, Renée De Jong, Mily Johana Ramírez Ruiz, Beatrice Nyamwenge Okech, Rose September, Anshu Mohan, Sarah Kline

**Affiliations:** Victoria Institute of Strategic Economic Studies, Victoria University, PO Box 14428, Melbourne, Victoria 8001, Australia; Centre for Mental Health and Community Wellbeing, Melbourne School of Population and Global Health, The University of Melbourne, 207 Bouverie Street, Carlton, Victoria 3010, Australia; Victoria Institute of Strategic Economic Studies, Victoria University, PO Box 14428, Melbourne, Victoria 8001, Australia; National Department of Health, Adolescent and Youth Health, Dr. AB Xuma Building, 1112 Voortrekker Rd, Pretoria Townlands 351-JR, Pretoria, South Africa; World Health Organization South-East Asia Regional Office, Family and Gender Health, World Health House, Indraprastha Estate, Mahatma Gandhi Marg, New Delhi 110 002, India; Ministry of Health and Social Protection, Directorate of Promotion and Prevention, Carrera 13 No. 32–76, Teusaquillo, Bogotá 111321, Colombia; Amref Health Africa, PO Box 30125, Wilson Airport, Nairobi 00100, Kenya; Accelerated Programme of Action for Children, The Presidency of the Republic of South Africa, Union Buildings, Government Avenue, Pretoria 0001, South Africa; World Health Organization South-East Asia Regional Office, Family and Gender Health, World Health House, Indraprastha Estate, Mahatma Gandhi Marg, New Delhi 110 002, India; United for Global Mental Health, 168 Shoreditch High St, London E1 6RA, United Kingdom

**Keywords:** adolescents, mental health, depression, anxiety, education, cost-benefit analysis

## Abstract

Adolescence is a critical period for the emergence of mental health disorders. The prevalence of depression and anxiety among adolescents in high- and middle-income countries has increased over the past three decades. According to the Global Burden of Disease Study (2025), mental disorders account for 21.4% of the adolescent burden of disease in high socio-demographic index (SDI) countries and 16.1% and 18.6% in the middle- and low-SDI countries. Despite the high and rising burden, adolescent mental health remains less prioritized in policy and funding. Treatment rates in low- and middle-income countries are low, with fewer than one in ten affected adolescents receiving care in some settings. Poor mental health in adolescence is associated with long-term losses in educational attainment, productivity and social participation. There is growing evidence suggesting that mental health interventions for depression and anxiety can be cost-effective even in resource constrained settings. This paper presents new evidence on the return on investment from scaling up interventions for adolescent depression and anxiety in two middle-income countries: Colombia and South Africa. Using the OneHealth tool and an economic model previously applied in global adolescent investment cases, we estimate health, education and economic benefits under a scale-up scenario. Scaling-up treatment for depression and anxiety in Colombia results in a benefit-cost ratio (BCR) for economic benefits alone of 13.9 and for combined economic and social benefits of 15.4. For South Africa the BCRs are 13.0 and 13.9, respectively. Our findings offer policy-relevant evidence to support increased investment in adolescent mental health in settings where data and resources remain limited.

Key messages

- The rate of mental disorders among adolescents increased between 1990 and 2021 for low- and middle-socio-demographic index (SDI) countries and especially for high SDI countries. The increase was particularly marked for the most prevalent mental disorders—anxiety and major depressive disorder—which are responsible for over half of the burden of mental disorders. Although the prevalence of mental disorders among adolescents is high and increasing, treatment rates are low, especially in resource-constrained settings. Scaling-up treatment for depression and anxiety among adolescents yields strong returns on investment. Our findings are policy-relevant evidence to support increased investment in adolescent mental health in settings where data and resources remain limited.
- Early investment in mental health would keep adolescents with depression and anxiety in school longer and result in greater productivity in the long run.
- Implementation is constrained by limited human resources. Governments and partners should invest in further studies to establish outcomes from training non-specialist providers, (school counsellors and community health workers) to deliver first-line mental healthcare especially in primary care and educational settings.
- Data gaps remain a major barrier to planning and resource allocation. National health information systems should be strengthened to include age-disaggregated data on adolescent mental health, treatment coverage, and outcomes to enable better tracking of progress and needs.

## Introduction

Although there have been marked improvements in several areas of adolescent health and well-being over recent decades, this has not been the case for mental health. The rate of mental disorders among adolescents reported in the Global Burden of Disease (GBD) 2023 Study (Global Burden of Disease Collaborative Network 2024, 2025) increased between 1990 and 2023 for low- and middle-socio-demographic index (SDI) countries and especially for high SDI countries. By contrast, the rate of all other causes declined (Table 1).

**Table 1:** Rates of mental disorders among adolescents in 2023 and percentage change, 1990–2023, persons aged 10–19 years.

	Prevalence rate per 100 thousand			Percentage change 1990–2021		
	High SDI	Middle SDI	Low SDI	High SDI	Middle SDI	Low SDI
All causes	91 752	95 393	97 293	−2.9	−3.4	−2.2
Mental disorders	19 630	15 337	18 080	35.7	25.9	40.2
All other causes	72 121	80 056	79 213	−9.9	−7.5	−8.5
Anxiety disorders	9114	6954	9083	82.4	61.7	123.1
Major depressive disorder	3204	2403	2968	50.5	46.3	32.2

SDI is socio-demographic index.

Source: Global Burden of Disease Collaborative Network (2024, 2025).

This increase in mental disorders in all country categories contrasts with the falls experienced by all other contributors to the burden of disease. The increase was particularly marked for the most prevalent mental disorders—anxiety and major depressive disorder—which are responsible for over half of the burden of mental disorders.

Mental disorders accounted for 21.4% of the adolescent burden of disease in 2023 in high SDI countries, and 16.1% and 18.6% in middle SDI and low SDI countries, respectively. In 2023, around 19.6% of adolescents in high SDI countries suffered from a mental disorder with somewhat lower rates in middle SDI (15.3%) and low SDI (18.1%) countries.

However, as the Lancet Commission on Adolescent Health and Wellbeing has argued, the relative lack of high-quality data in low- and middle-income countries (LMICs) may underestimate the true levels of mental disorder among adolescents in these countries (Baird et al. 2025).

In their systematic review of the prevalence of mental health problems in sub-Saharan adolescents, Jörns-Presentati et al. (2021) reported a median point prevalence for depression of 26.9% and 29.8% for anxiety. In contrast, however, recent surveys of adolescent mental health in Kenya, Indonesia, and Vietnam reported the prevalence of any mental disorder in the past 12 months to be 12.1%, 5.5%, and 3.3%, respectively (Erskine et al. 2024).

Around three quarters of lifetime mental disorders have their onset before age 24 years (Erskine et al. 2024), so prevention and the treatment during adolescence are important across the lifecycle. In addition, mental health is explicitly recognized as a crucial component of children's rights within the United Nations Convention on the Rights of the Child.

In this paper, first we review the evidence on the economic benefits of interventions addressing adolescent mental health as shown in previous return on investment (ROI) studies. We then present the results from studies of the ROI from interventions to reduce depression and anxiety among adolescents in two middle-income countries: Colombia and South Africa, undertaken as part of a larger study of adolescent health and well-being conducted by the Victoria Institute for Strategic Economic Studies with the Partnership for Maternal, Newborn, and Child Health and partners (WHO 2024).

### Causes

The causes of depression and anxiety among adolescents are varied and include biological, environmental, and psychological factors. Risk factors for adolescent depression include female gender, family history of depression, personal history of trauma, chronic medical illness, family conflict, and minority sexual orientation (Miller and Campo 2021). Inherited risks, developmental factors, sex hormones, and psychosocial adversity interact to increase risk through hormonal factors and associated perturbed neural pathways (Thapar et al. 2012).

In their review of anxiety among adolescents, Anderson et al. (2025) identified six current trends that contribute to the increasing prevalence of anxiety among youth, including biological aspects, the rise of digital technology and social media, changes in nuclear family dynamics, academic pressures, and political and environmental uncertainty.

The current generation of adolescents is uniquely vulnerable when compared to previous generations, due to their exposure and consumption of knowledge via social media, and increased stressors from other aspects of life (Anderson et al. 2025). However, the Lancet Commission on Adolescent Health and Wellbeing notes that evidence for the effect of digital media—and particularly social media—on adolescent mental health is highly contested (Baird et al. 2025).

### Treatment rates

Although the prevalence of mental disorders among adolescents is high and increasing, treatment rates are low.

Wang et al. (2023b) conducted a review and meta-analysis of 40 studies from 14 countries of treatment rates for mental disorders among children and adolescents, and found that the pooled treatment rate was 38% for any mental disorder, 36% for depressive disorders, 31% for anxiety disorders, 58% for attention deficit hyperactivity disorder, and 49% for behaviour disorders. The treatment rate for any mental disorder in lower middle-income countries was 6%, in upper-middle-income countries (UMICs) was 24%, and in high-income countries (HICs) was 43%.

Data from three national surveys suggest that only 11% of children and adolescents in Kenya, 8.4% in Viet Nam, and 2.6% in Indonesia receive treatment for their mental disorders (Baird et al. 2025).

#### Colombia

The most recent Colombian survey for which data is available is the Colombian National Mental Health Survey (Encuesta Nacional de Salud Mental) (MSPS 2015), which reported that 43% of adolescents aged 12–17 with a mental illness received treatment.

However, Gómez-Restrepo et al. (2023), citing Gómez-Restrepo et al. (2016) and Thornicroft et al. (2017), assert that less than 12% of adolescents in LMICs such as Colombia receive even minimally adequate treatment, pointing to an issue of both access and quality.

Thornicroft et al. (2017) reported the results from representative community household surveys from 21 countries as part of the World Health Organization World Mental Health Surveys. For Medellin in Colombia, they found that 9% of those people with major depressive disorders received minimally adequate treatment. Across major cities in Colombia, it was 5%. South Africa was not included.

These studies suggest that the coverage rate for treatment of depression and anxiety among adolescents in Colombia is between 5% and 43% with middle values of 9%–12%.

Wang et al. (2023b) conducted a review and meta-analysis of 40 studies from 14 countries of treatment rates for mental disorders among children and adolescents and concluded that the treatment rate for children and adolescents in UMICs such as Colombia and South Africa was 13%.

#### South Africa

According to Docrat et al. (2019), crude estimates indicate that only 0.89% and 7.35% of the uninsured population of South Africa requiring care received some form of public inpatient and outpatient mental healthcare, respectively in 2016–17. Further, mental health human resource availability, infrastructure, and medication supply are significant constraints to the realization of the country’s progressive mental health legislation.

Simelane et al. (2025) assert that an estimated 17% of South Africa’s children have a diagnosable and treatable mental disorder, yet only one in 10 are able to access care, with specialist child and adolescent mental health services being concentrated in a handful of urban centres.

### Treatments for depression and anxiety

The standard treatments for depression and anxiety are psychosocial interventions such as cognitive behavioural therapy (CBT) and, in certain circumstances (see below), antidepressant medicines.

A systematic review and meta-analysis by Cuijpers et al. (2023) of psychotherapies in the treatment of depression in children and adolescents, found that there was a recovery rate of 58% compared to 36% rate in controls. However, more than 60% of youth receiving therapy did not respond. Clinician-delivered CBT and interpersonal psychotherapy can be effective in reducing symptoms of both depression and anxiety across a range of settings, including schools, community clinics, and specialist services (Oud et al. 2019, Baker et al. 2021). However, access to these interventions can be limited by workforce shortages and service availability, especially outside urban centres.

Antidepressants have been shown in trials to be effective for treating depression in adolescents, with a meta-analysis suggesting that the number needed to treat for 1 youth to have a response is 10 (Miller and Campo 2021). The landmark Treatment for Adolescents with Depression Study showed that fluoxetine was significantly superior to placebo (response rate, 61% versus 35%) at week 12 of treatment (Miller and Campo 2021). However, antidepressants are not recommended as a first-line treatment and must be prescribed with caution due to concerns about side effects, including increased risk of suicidal ideation in a minority of young people, particularly in the early stages of treatment. WHO guidelines recommend that fluoxetine (a selective serotonin reuptake inhibitor antidepressant) is only considered alongside psychological therapies in adolescents aged 13 years or more who have not responded to psychological therapies on their own, and only if careful monitoring is possible (WHO 2023).

Because a majority of adolescents are most likely to be in education, attention has turned to school-based interventions targeting mental health (WHO 2023).

A meta-analysis by Zhang et al. (2023) of 29 studies of 32 programmes concluded that school-based mental health promotion interventions are effective at reducing depression and anxiety with best results from CBT delivered through clinicians at the secondary school level. This review confirmed a more comprehensive earlier systematic review and meta-analysis that included 158 studies and concluded that ‘universally delivered interventions can improve adolescent mental health and reduce risk behavior’ (Skeen et al. 2019).

Simelane et al. (2025) have argued that schools in South Africa are well placed to play a pivotal role in supporting the mental health of children and adolescents, although they are overburdened with limited capacity to provide comprehensive mental health support.

A number of studies have examined the impact of internet-based programmes for addressing adolescent mental health. A systematic review and meta-analysis by Ma et al. (2024) of 23 randomized controlled trials indicated that internet-based self-help therapies significantly reduced depression in adolescents and young adults. Interventions lasting 30–60 min, and 60–180 min per week were effective in the short term. However, factors such as patient recruitment locations, medication usage, therapists’ involvement, weekly intervention time, and intervention duration interacted with the outcome.

A similar review by Wang et al. (2023a) of randomized controlled trials investigated internet-based self-help interventions aiming to mitigate mental health symptoms such as anxiety and depression. In comparison to controls, participants receiving interventions reported significant reductions in symptoms of anxiety, depression, and stress, along with a significant improvement in quality of life. Longer interventions were particularly effective in reducing anxiety and depression. Additionally, third-wave CBT showed pronounced intervention effects. Self-guided interventions tended to show smaller effect sizes and higher dropout rates, though they offered benefits for mild symptoms or for adolescents reluctant to engage in traditional services.

LaMonica et al. (2025) argue that digital health technologies have the potential to play a vital role in increasing equity of access to care and improving mental health and functioning.

### Return on investment studies

There have been a number of studies that have presented an investment case for interventions to address mental disorders.

Chisholm et al. (2016) examined a global investment case for a scaled-up response to the public health and economic burden of depression and anxiety disorders. They used the mental health module of the OneHealth tool (OHT) (WHO 2025) to calculate treatment costs and health outcomes in 36 countries between 2016 and 2030. Across country income groups, resulting BCRs were estimated to be 2.3–3.0 to 1 (i.e. a return of 2.3–3.0 US dollars for every dollar invested) when economic benefits only were considered, and 3.3–5.7 to 1 when the value of health returns was also included.

In a recent study, Chisholm et al. (2023) used the OHT to undertake national mental health investment case studies in Bangladesh, Kenya, Nepal, Philippines, Uganda, Uzbekistan, and Zimbabwe. They found that for most conditions and country contexts there were benefit cost ratios of up to 10.6 when productivity gains alone were included, and up to 30.3 when the intrinsic economic value of health was also considered. There was considerable variation in benefit-cost ratios between intervention areas, with population-based preventive measures and treatment of common mental and neurological conditions showing the most attractive returns when all assessed benefits are taken into account.

More recently, Herbig et al. (2025a, 2025b) observed that mental health conditions only receive 2% of domestic government healthcare funding globally, resulting in an annual funding gap of US$200 billion to US$350 billion. Using the OHT and other modelling resources, they estimated that scaling up known, cost-effective interventions to prevent, treat, and help people recover from mental health conditions could reduce the burden of these conditions with 150 million disability adjusted life years (DALYs) averted globally in 2050, with each $1 invested in scaling up mental health interventions having an economic return of $5–$6.

This value is similar to that estimated in the Chisholm et al. (2016) study. Unlike that study, the Herbig et al. (2025a) study also included benefits arising from non-communicable disease prevented by interventions addressing mental health, as well as the educational benefits from increased school attendance among adolescents.

Stelmach et al. (2022) conducted an economic modelling study to quantify the potential costs and benefits of mental health interventions to prevent or treat anxiety, depression, bipolar disorder, and suicide among adolescents. They developed a Markov model that followed cohorts of adolescents (ages 10–19 years) from 36 countries Implementing the selected interventions resulted in an estimated ROI of 23. The high ROI was observed across regions and country income levels, with the highest ROI arising from treating mild depression with group-based CBT, prevention of suicide attempts among high-risk adolescents, and universal prevention of combined anxiety and depression in low-income and lower middle-income countries.

The interventions for anxiety and depression modelled by Stelmach et al. (2022) included CBT delivered individually in person, in a group in person, and individually using an internet-based communication platform. They also included an internet-based guided self-help intervention, based on the principles of CBT delivered through self-guided modules. The interventions for preventing depression included a step to screen adolescents and identify those with elevated symptoms, suggesting they either have or are at high risk of developing major depression. Treatment for major depression included individual in-person CBT and the addition of fluoxetine.

A study by Thomas et al. (2026) modelled the impact of 12 interventions to prevent depression and anxiety among Australian adolescents using data on sociodemographic risk factors that were relevant for the included interventions (bullying, financial stress, poor school connectedness, racial discrimination, child maltreatment). The BCRs of interventions varied widely depending on whether they impacted depression and anxiety directly or indirectly through reduction of a risk factor with BCRs ranging from 5.1–19.3.

## Methods

Despite their different histories, South Africa and Colombia have perhaps surprising similarities. Their economies, with a gross domestic product (GDP) of US$400 billion and US$410 billion and populations, 64 million and 53 million in 2024, for South Africa and Colombia, respectively, are not dissimilar (World Bank 2025c). In other ways, however, they are moving in different directions with South Africa’s population growing younger, while Colombia’s ages (World Bank 2025c). The number of adolescents in Colombia is projected to decline from 7.2 million in 2024 to 7.0 million in 2040, while South Africa’s is expected to increase from 10.7 million in 2024 to 11.8 million by 2040 (World Bank 2025c). Adolescents in both countries suffer from a high level of interpersonal violence and above global average levels of mental disorders. It is likely that the two are related (Cluver et al. 2018, Vibhakar et al. 2019, Unidad para las Víctimas 2024). Measured by burden of disease in 2023, adolescents in Colombia and South Africa had mental disorder prevalence rates of 14 467 per 100 000 and 19 071 per 100 000 persons, respectively (Global Burden of Disease Collaborative Network 2025).

Using an approach similar to that of Chisholm et al. (2016), we modelled interventions to reduce depression and anxiety among adolescents in Colombia and South Africa using the OHT (WHO 2025). The results from the OHT modelling were then used in an economic model to undertake an ROI analysis.

### Interventions and their coverage

The OHT includes nine interventions used in the treatment of mild, moderate, and severe depression and anxiety, and these are listed in Table 2. Detailed descriptions of these interventions, as well as types of antidepressant and psychosocial treatments, providers, and settings, can be found in the OneHealth Intervention Assumptions manual (Avenir Health 2016).

**Table 2:** Adolescent-relevant depression and anxiety OHT interventions, base, and scale-up coverage rates, Colombia and South Africa.

Intervention	Base	Scale-up	Scale-up
		Colombia	South Africa
Anxiety disorders			
Basic psychosocial treatment for anxiety disorders (mild cases)	10	35	45
Basic psychosocial treatment and anti-depressant medication for anxiety disorders (moderate-severe cases)	10	35	45
Intensive psychosocial treatment and anti-depressant medication for anxiety disorders (moderate-severe cases)	10	35	45
Depression			
Basic psychosocial treatment for mild depression	10	35	45
Basic psychosocial treatment and anti-depressant medication of first episode moderate-severe cases	10	35	45
Intensive psychosocial treatment and anti-depressant medication of first episode moderate-severe cases	10	35	45
Intensive psychosocial treatment and anti-depressant medication of recurrent moderate-severe cases on an episodic basis	10	35	45
Intensive psychosocial treatment and anti-depressant medication of recurrent moderate-severe cases on a maintenance basis	10	35	45
Psychosocial care for peri-natal depression	10	35	45

OHT is OneHealth tool.

Source: WHO (2025).

The modelling approach described below relies on comparing the benefits and costs from two scenarios (base and scale-up) with an intervention period of 2024–35. This requires specifying the baseline coverage rate in 2024 and the scale-up coverage rate in 2035, where the coverage rate means the proportion of people needing treatment who receive it.

For the base scenario, coverage rates are assumed to be unchanged from their 2024 levels across the period. For the scale-up scenario, coverage rates for the years between 2024 and 2035 are interpolated using the linear interpolation setting within the OHT. The cost and impact of interventions are, therefore, modelled as a result of changing treatment coverage rates.

Chisholm et al. (2023) used the OHT to model the impact of interventions to reduce depression and anxiety in 7 countries. Bangladesh, Uganda, and Uzbekistan were modelled over a 10-year period. Kenya, Nepal, Philippines, and Zimbabwe were modelled over a 20-year period. The coverage rates used in the modelling are reproduced in Table 3.

**Table 3:** OHT intervention coverage rates, baseline and target, by mental, neurological, and substance use condition, %.

	Baseline	Target	Target/baseline
Anxiety			
** 10-year period**			
Bangladesh	15	58	3.9
Uganda	10	48	4.8
Uzbekistan	4	29	7.3
** 20-year period**			
Kenya	8	48	6.0
Nepal	10	48	4.8
Philippines	10	48	4.8
Zimbabwe	11	48	4.4
Depression			
** 10-year period**			
Bangladesh	11	50	4.5
Uganda	11	50	4.5
Uzbekistan	3	32	10.7
** 20-year period**			
Kenya	8	50	6.3
Nepal	8	50	6.3
Philippines	8	50	6.3
Zimbabwe	9	50	5.6

Source: Chisholm et al. (2023).

The country most similar regionally to South Africa with a 10-year scale-up period is Uganda. The assumed baseline coverage rate is 10% for anxiety and 11% for depression. The target coverage rates are 4.8 and 4.9 times the baseline rates.

With a 20-year scale-up period, the countries most similar to South Africa are Kenya and Zimbabwe with baseline rates of 8% and 11% for anxiety and 8% and 9% for depression, respectively.

None of the countries in this study are from South America. Perhaps the closest comparison with Colombia would be the Philippines where Spanish is also common. The baseline coverage rates for anxiety and depression for the Philippines are 10% and 8%, respectively.

### Colombia

The review of earlier studies indicates a baseline coverage rate of 12%–13%. The values for Colombia above suggest baseline coverage rates of 8%–10%. Based on this, we apply a 10% baseline coverage rate across all interventions and a target rate of 35% for depression and anxiety for adolescents in Colombia (Table 2).

### South Africa

The review of studies indicates a coverage rate of between 7.35% and 10%. Based on this and the discussion earlier, we apply a 10% baseline coverage rate across all interventions and a target rate of 45% for depression and anxiety for adolescents in South Africa (Table 2 covers both Colombia and South Africa).

#### Cost

The approach to estimating the costs associated with the interventions is similar to that used in the investment case for scaling up treatment of depression and anxiety (Chisholm et al. 2016), as well as for the UNFPA global adolescent health study (Sheehan et al. 2017, Sweeny et al. 2019) and a study on reproductive, maternal, newborn, and child health (Stenberg et al. 2014).

The OHT includes cost assumptions for the resource inputs needed to provide health services in Colombia and South Africa. From OHT, estimates were obtained by year of the costs of drugs and supplies, the number of inpatient days, the number of outpatient visits, and the amount of personnel time in minutes.

The cost of inpatient days was calculated by multiplying the number of days by the WHO CHOosing Interventions that are Cost Effective (CHOICE) estimates of the average cost per bed day for Colombia and South Africa (WHO 2021) expressed in US dollars at 2023 values. Similarly, the cost of outpatient visits was estimated by multiplying the estimated number of outpatient visits by the WHO CHOICE estimates of the average cost per outpatient visit for Colombia and South Africa (WHO 2021). The cost of personnel time was calculated by multiplying the personnel time in person-years by WHO CHOICE estimates of the average annual salaries for physicians, nurses, and other health workers in Colombia and South Africa expressed in US dollars at 2023 values (Serje et al. 2018).

The other components of cost: supply chain, infrastructure and equipment, governance, health information systems, health financing, emergency and reconstruction, and additional programmes, were calculated as a markup of the combined direct costs of drugs and supplies, inpatient days, outpatient visits, and personnel time. Results from a study that looked at the investment needed to advance the UHC agenda in 67 countries for the period 2016–30 (Stenberg et al. 2017), indicated that the total investment cost was, on average, 2.2–2.5 times the combined direct costs. As adolescents account for around 16% of the population of Colombia and South Africa, some of the costs included within these estimates are not likely to be applicable for investments in health services for adolescents. The total costs were therefore estimated as two times the combined direct costs.

#### Health outcomes

When extracting health outcomes results for depression and anxiety, we used the ‘Impact scale-up setting’ and ‘By age groups’ settings within OHT to obtain results showing the impact of the scale-up scenario compared to the base scenario for adolescents.

Over the period 2024–35, the reduction in the number of cases of depression and anxiety in Colombia due to the interventions was estimated to be 29 979 for females and 19 613 for male adolescents. For South Africa, the reduction in the number of cases of depression and anxiety due to the interventions was estimated to be 76 177 for female and 66 157 for male adolescents (Table 4).

**Table 4:** Reduction in the number of cases of depression and anxiety among adolescents, 2024–35.

Condition	Colombia		South Africa	
	Female	Male	Female	Male
Depression	16 743	11 467	42 605	39 975
Anxiety	11 001	8146	28 486	26 182
Perinatal depression	2235	—	5086	—
Total	29 979	19 613	76 177	66 157

Source: Authors’ calculations.

A number of articles have been published about the estimates for the GBD 2021 (Global Burden of Disease Collaborative Network 2024) provided by the Institute for Health Metrics at the University of Washington. These include a discussion of the methods used to estimate the disability weights used in the calculation of years lived with disability and DALYs (Ferrari et al. 2024). For depression and anxiety, the GBD descriptions of severity states, their estimates for the disability weights and the proportion of mild, moderate, and severe cases are shown in Table 5.

**Table 5:** Disability weights and proportion of cases, depression and anxiety, GBD 2021.

Severity	Description	Disability weight	Proportion
Depression			
Mild	Feels persistent sadness and has lost interest in usual activities. The person sometimes sleeps badly, feels tired, or has trouble concentrating but still manages to function in daily life with extra effort.	0.145	68.6
Moderate	Has constant sadness and has lost interest in usual activities. The person has some difficulty in daily life, sleeps badly, has trouble concentrating, and sometimes thinks about harming himself (or herself).	0.396	19.8
Severe	Has overwhelming, constant sadness and cannot function in daily life. The person sometimes loses touch with reality and wants to harm or kill himself (or herself).	0.658	11.6
Anxiety			
Mild	Feels mildly anxious and worried, which makes it slightly difficult to concentrate, remember things, and sleep. The person tires easily but is able to perform daily activities.	0.030	50.9
Moderate	Feels anxious and worried, which makes it difficult to concentrate, remember things, and sleep. The person tires easily and finds it difficult to perform daily activities.	0.133	29.8
Severe	Constantly feels very anxious and worried, which makes it difficult to concentrate, remember things, and sleep. The person has lost pleasure in life and thinks about suicide.	0.523	19.3

GBD is Global Burden of Disease.

Source: Ferrari et al. (2024).

Beck et al. (2011) surveyed 771 patients in the USA using the Patient Health Questionnaire 9-item screen, a measure of depression symptom severity, and the Work Productivity and Activity Impairment questionnaire, to measure loss in productivity due to absenteeism and presenteeism according to severity of depression. They found that productivity loss increased with severity as shown in Table 6. Although Beck et al. (2011) report productivity loss of 51.3% for severe depression, the definition of severe depression and anxiety in Table 5 suggests that the productivity losses for severe depression and anxiety are likely to be 100% and these values are used in subsequent analysis in this paper.

**Table 6:** Productivity loss, %, and severity of depression.

Severity	PHQ-9 scores	Loss in productivity
Mild	7–9	29.6
Moderate	10–14	38.0
Severe	20+	51.3

PHQ-9 is Patient Health Questionnaire 9-item screen.

Source: Beck et al. (2011).

The proportions of cases of depression and of anxiety with the different degrees of severity used in the GBD 2021 are shown in Table 5. Applying these to the total number of cases of depression and anxiety reduced by the package of interventions outlined in Table 2, gives the reduction in severe, moderate, and mild depression and anxiety for females and males shown in Table 7.

**Table 7:** Reduction in the number of cases of depression and anxiety among adolescents, by severity, 2024–35.

Condition	Colombia		South Africa	
	Female^a^	Male	Female^a^	Male
**Severe**				
Depression	2207	1333	5533	4636
Anxiety	2126	1574	5499	5054
**Moderate**				
Depression	3752	2267	9442	7915
Anxiety	3275	2426	8488	7802
*Mild*				
Depression	13 020	7867	32 716	27 423
Anxiety	5600	4146	14 499	13 326

^a^For females, depression includes perinatal depression.

Source: Authors’ calculations.

### Economic and social benefits

To estimate the ROI for health intervention programmes, it is necessary to express the improved health outcomes in economic terms and compare these with the costs of the interventions. As noted above, the approach builds on previous studies (Sheehan et al. 2017, Sweeny et al. 2019) focused on economic and social benefits.

#### Economic benefits

Comparing an intervention scale-up scenario with a base scenario enables the number of deaths and amount of morbidity averted to be calculated and compared to the additional cost. Within our model, we consider that the economic benefits of interventions preventing deaths occur when people who would otherwise die prematurely are in the workforce and produce economic output during their working life. A similar benefit occurs for people who would otherwise suffer a serious disability that prevents them from working.

In this study, the economic modelling of mortality follows the cohort of deaths averted for each of the years from 2024 to 2035. Each cohort was classified by age and sex. As the cohort ages, it is subject to the mortality rates applicable to that age group, sex, and year based on estimates from the UN World Population Prospects data (2024) for Colombia and South Africa. The effect of avoided mortality on the labour force was calculated by taking the numbers of deaths avoided by age and gender, and applying a corresponding labour force participation rate for this age, gender and year sourced from the International Labor Organization (ILO) projections of labour force participation rates (ILO 2025) for Colombia and South Africa.

The contribution that each of these labour force cohorts makes to economic output was calculated by multiplying the number in each age and sex category by a productivity level that varies with age and year. To do this, the average productivity was first calculated by dividing the World Bank estimate of GDP in current US dollars by the labour force for the most recent year (2022) for which data were available (World Bank 2025a). This average productivity was then increased at an annual rate determined by the country’s income status. The methodology for this is described in more detail in Sweeny et al. (2019).

Productivity varies by age so, as a proxy for this, the distribution of hourly wage rates by age for Australia for 2023 (ABS 2025) was used, as there is no comparable data for Colombia or South Africa. Average productivity for each age group was calculated by multiplying average productivity by the ratio of hourly wage rates for the age group to overall hourly wage rates. The total GDP generated was calculated by summing up the GDP produced by each cohort for each year of the period in which they would be in the labour force. The contribution to GDP of each cohort of persons who would otherwise suffer from serious disabilities was calculated in a similar way as for mortality, using the same assumptions about participation rates and productivity.

According to the GBD 2021 (Ferrari et al. 2024), the average duration of a depressive episode is assumed to be 0.65 of a year, or 7.8 months. We have assumed that this is also the case for anxiety and is the same for males and females.

For severe depression and anxiety based on their definitions in Table 4, we assume no labour force participation during the length of an episode. For moderate and mild episodes, we assume the productivity loss while employed during an episode as reported in Table 5.

The number of persons in the labour force is calculated by applying the ILO labour participation rate projections for 15–19-year-old males and females for Colombia and South Africa to the reduction in depression and anxiety (Table 6). Average productivity in Colombia and South Africa is estimated by dividing the World Bank estimate of GDP in US dollars (World Bank 2025a) by the ILO estimate of the total labour force (ILO 2025). Projections over the period 2024–35 were obtained by applying the projected mean long-term annual productivity growth rate for UMICs.

For each level of depression and anxiety, the productivity is adjusted by the assumed productivity loss.

Summing across levels of severity gives the economic benefit measured as the contribution to GDP from reduced depression and anxiety for each year of the period.

#### Social benefits

Health has an intrinsic value and, in addition to the market value put on health improvements (here captured by labour productivity), many studies apply a ‘value of a statistical life’ (VSL) approach. This allows researchers to fully capture the value of the social benefit of health improvement.

Stenberg et al. (2014) applied an approach in which benefits not captured in labour productivity measures were referred to as ‘social benefits’. The social benefits were estimated within a VSL envelope that was, on average, assuming the value of a life year as 1.5 times GDP per capita. As the calculated economic benefit in the Stenberg et al. (2014) study represented one times GDP per capita, this left a residual value of 0.5 times GDP per capita as the estimated social benefit. Following the same approach, a value of 0.5 GDP per capita was assigned to each DALY gained from the interventions to estimate the social benefit of improved health.

### Education outcomes and benefits

The benefits described in the previous section are based on the increased productivity of adolescents in the labour force as a result of the interventions.

Most adolescents, however, will still be in education, so this section estimates the economic benefits arising from increased school attendance as a result of the interventions. In 2022, 92% of students in South Africa completed primary school (World Bank 2025b). In South Africa, the average age for completing secondary school is between 17 and 18 years (OECD 2023) with 63% of adolescents completing upper secondary school in 2021 (UNESCO 2025).

According to the ILO (2025), the labour force participation rates for the age group 15–19, is 8.2% for females and 9.9% for males.

The World Bank estimates that 4.4% of adolescents in South Africa are neither in the labour force nor in education (World Bank 2025b). This means that 87.4% of females and 85.7% of males are in education.

In 2022, 97.1% of students in Colombia completed primary school (World Bank 2025b, 2025c). In Colombia, the average age for completing secondary school is between 16 and 17 years old (OECD 2023). According to the ILO (2025), the labour force participation rate for the age group 15–19, is 23.6% for females and 36.1% for males. The World Bank estimates that 4.4% of adolescents in Colombia are neither in the labour force nor in education (World Bank 2025b). This means that 72.0% of females and 59.5% of males are in education.

Based on the analysis by Esch et al. (2014), Stelmach et al. (2022) estimated the increased odds of failing to complete secondary education among adolescents with anxiety in secondary school as 1.40 and with depression in secondary school as 3.38. This suggests that adolescents suffering from anxiety or depression will not achieve the same numbers of years of schooling as those without anxiety or depression.

In a study of an education and development investment case for Syria, Rasmussen et al. (2022) found that based on the findings of Lawrence et al. (2019), mental disorders would result in a loss of 13% of days at secondary school. These results for Australia are likely to be high compared to Colombia and South Africa, where the mental disorder burden is approximately 75% of the Australian level (Global Burden of Disease Collaborative Network 2024).

The average years of secondary schooling in Colombia is 5.4 years and in South Africa are 5.0 years, so the loss in time in school, based on these findings, would be equal to about half of a year in secondary school (World Bank 2025b). In their analysis of the returns to investment in education, Psacharopoulos and Patrinos (2018, Annex 2) estimate that an extra year of secondary schooling will increase earnings by 11.3% in Colombia and 23.0% in South Africa. For an extra half of a year, this increase would be 5.7% for Colombia and 11.5% for South Africa.

Using the estimated numbers of cases of depression and anxiety averted arising from the OHT modelling (Table 7), we calculate the number of those in education using the estimates above for the percentage in school.

The economic benefit from the extra amount of schooling for these adolescents is calculated in the same way as described above.

## Results

The ROI can be expressed in a number of different, but related ways. This study reports on BCRs, as they are intuitively easy to understand. The BCR divides the estimate of benefits by the estimate of costs. A BCR >1 means that the benefits of an intervention programme are greater than the costs of the programme.

It is standard practice when calculating BCRs to express these benefits and costs in net present value terms. Because benefits and costs for years in the future are usually regarded as having a lower value than those in the present, a discount rate is applied to these future benefits and costs. Most analyses of long-term projects adopt the standard World Bank discount rate of 3% to calculate NPVs (Campos et al. 2015). This approach has been used in this study, though a sensitivity analysis has also been carried out assuming discount rates of 0%, 2%, and 5%, as well as 3%.

Tables 8 and 9 show the economic and social benefits and costs calculated for a range of discount rates and associated BCRs for Colombia and South Africa, respectively. Benefits are lifetime estimates and costs are for the period 2024–35.

**Table 8:** Depression and anxiety among adolescents in Colombia, economic and social benefits, costs, and BCRs, various discount rates.

	0%	2%	3%	5%	0%	2%	3%	5%
	US$ millions				COP billions			
Economic benefits, morbidity	49.2	41.4	38.1	32.3	205.2	172.6	158.9	134.7
Economic benefits, mortality	36.9	17.9	12.9	7.1	153.9	74.6	53.8	29.6
Economic benefits, education	1355.2	677.4	510.6	310.9	5651.2	2824.8	2129.2	1296.5
Total economic benefits	1441.3	736.6	561.5	350.3	6010.2	3071.6	2341.5	1460.8
Social benefit	85.7	64.3	57.5	47.2	357.4	268.1	239.8	196.8
Cost	51.4	43.6	40.3	34.5	214.3	181.8	168.1	143.9
BCR—economic	28.0	16.9	13.9	10.1	—	—	—	—
BCR—economic plus social	29.7	18.4	15.4	11.5	—	—	—	—
Education only								
BCR—economic	26.4	15.5	12.7	9.0	—	—	—	—

COP is Colombian pesos, exchange rate is 4170 COP per US$. BCR is benefit-cost ratio.

Source: Authors’ calculations.

**Table 9:** Depression and anxiety among adolescents in South Africa, economic and social benefits, costs, and BCRs, various discount rates.

	0%	2%	3%	5%	0%	2%	3%	5%
	US$ millions				Rand millions			
Economic benefits, morbidity	195.3	164.3	151.0	128.1	3633.2	3055.2	2808.0	2382.5
Economic benefits, mortality	110.2	57.1	42.1	23.9	2049.0	1061.2	782.1	443.8
Economic benefits, education	5619.0	2911.8	2146.7	1218.8	104 513.6	54 158.7	39 928.0	22 670.5
Social benefit	247.3	188.1	168.1	137.7	4600.6	3499.2	3126.3	2561.2
Cost	230.1	195.3	180.4	154.6	4279.7	3633.2	3355.4	2874.9
BCR—economic	25.7	16.0	13.0	8.9	—	—	—	—
BCR—economic plus social	26.8	17.0	13.9	9.8	—	—	—	—
Education only								
BCR—economic	24.4	14.9	11.9	7.9	—	—	—	—

Exchange rate is 18.6 Rand per US$. BCR is benefit-cost ratio.

Source: Authors’ calculations.

For Colombia, at a discount rate of 3% and assuming 0.5 years loss of schooling, the economic benefits from the interventions for depression and anxiety modelled using OHT are US$38.1 million (Colombian pesos (COP) 158.9 billion) for morbidity averted, US$12.9 million (COP 53.8 billion) for mortality averted, and US$510.6 million (COP 2129.2 billion) for improved education. The total economic benefits come to US$561.5 million (COP 2341.5 billion). The social benefits are US$57.5 million (COP 239.8 billion), and the cost is US$40.3 million (COP 168.1 billion). The estimate of the BCR for economic benefits alone is 13.9 and for combined economic and social benefits is 15.4. For the 49 592 adolescents treated over the period 2024–35 (Table 3), the cost per adolescent treated is US$812.6 (COP 3.389 million).

For South Africa, at a discount rate of 3% and assuming a 0.5-year loss of schooling, the economic benefits from the interventions for depression and anxiety modelled using OHT are US$151.0 million (2808.6 million Rand) for morbidity averted, US$42.1 million (782.1 million Rand) for mortality averted, and US$2146.7 million (39 928.0 million Rand) for improved education. The total economic benefits sum to US$2339.8 million (43 518.1 million Rand). The social benefits are US$168.1 million (3126.3 million Rand), and the cost is US$180.4 million (3354.4 million Rand). The estimate of the BCR for economic benefits alone is 13.0 and for combined economic and social benefits is 13.9. For the 142 334 adolescents treated over the period 2024–35 (Table 3), the cost per adolescent treated is US$1267.4 (23 574.4 Rand).

Despite the different demographic, economic and social profiles for Colombia and South Africa, the BCRs are quite similar. The substantial difference in total costs reflects mostly the higher number treated in South Africa than Colombia, but also differences in the costs of health care, such as hospital day costs, personnel costs, and the costs of supplies in the two countries. The total benefits are much higher in South Africa, reflecting a range of factors, including the number treated. Benefits in terms of lives saved and morbidity avoided are significantly higher than Colombia, but not sufficient to offset the higher costs in South Africa. The education benefits are relatively high for Colombia, given the lower treatment numbers, reflecting different returns to education.

## Discussion

The significant ROI results from the cases studies for Colombia and South Africa reinforce the findings from the other studies on the economic and social benefits arising from treatments for adolescent depression and anxiety such as Chisholm et al. (2016) and Herbig et al. (2025a). They provide evidence of the benefits of scaling up low levels of treatment in these two UMICs. These national studies also show the significant educational and lifetime earnings benefits from treating adolescents while in school, as suggested by the studies by Stelmach et al. (2022) and Thomas et al. (2026).

The BCRs reported in Tables 8 and 9 for interventions to treat depression and anxiety among adolescents are lower than those for interventions in other areas of adolescent well-being. Treating an episode of depression or anxiety does not prevent future episodes, so the benefits are more restricted than those for interventions that prevent death or serious disability, such as road safety programmes or vaccination against a variety of conditions, where benefits can be lifelong.

Because Colombia and South Africa differ in their demographic, economic and social profiles, the modelling results in different estimates for health outcomes, costs, and economic and social benefits. The two countries have different rates of prevalence for depression and anxiety and population size which means that the reduction in cases from the same scale up of interventions will differ. The costs of health care are determined by hospital day costs, personnel costs and the costs of supplies which influence cost outcomes. Similarly, the two countries have different sizes of GDP, labour force, labour force participation rates, and productivity levels leading to different levels of economic benefit. The demographic profiles of the two countries influence the estimates of GDP per capita and hence the social benefit estimates.

The ROI results for Colombia and South Africa differ from those in the other ROI studies discussed earlier (Table 10). In comparing the ROI results found in the current study to the study by Chisholm et al. (2016), it should be noted that the latter did not differentiate between adolescents and adults, and covered 36 countries. While the study by Stelmach et al. (2022), which covered adolescents in 36 countries, reported an average ROI of 23.6, they reported a large range of ROIs depending on the types of interventions modelled. For example, for treatment of mild depression using group CBT, they reported an ROI of 1692.6. The study by Herbig et al. (2025a) across broadly defined global regions, nonetheless, reports similar ROIs to the ones for Colombia and South Africa, as does the study of Australian adolescents reported by Thomas et al. (2026).

**Table 10:** Return on investment results from studies referenced.

Study	Countries included	ROI	Interventions
Chisholm et al. (2016)	36 countries	2.3–3.0	Treatment of depression and anxiety using CBT and medication
Herbig et al. (2025a)	Lower income country group, higher income country group	5–6	Interventions to prevent, treat and help people recover from mental health conditions
Stelmach et al. (2022)	36 countries	23.6	Treatment of depression and anxiety using CBT and medication
Thomas et al. (2026)	Australia	5.05–19.32	Preventive interventions for depression and anxiety
This study	Colombia	15.4	Treatment of anxiety and depression
This study	South Africa	13.9	Treatment of anxiety and depression

ROI is return on investment. CBT is cognitive behavioural therapy.

Sources: As listed in table.

Although there is less evidence of the benefits from school-based interventions and internet-based services, the results reported here suggest the need for further research to enable robust analyses of the economic and social benefits from these emerging ways of reducing the burden of poor mental health among adolescents.

Modelling necessarily requires assumptions about a range of parameters. While many of these are based on robust evidence, some such as treatment rates and the impact of different levels of severity of depression and anxiety on productivity, would benefit from further research. The impact of race, ethnicity, religion, income, geography, gender identity, sexual orientation, and disability on the transition to work by adolescents with depression and anxiety also needs additional scrutiny (Doan et al. 2024).

While the results reported here support scaling up mental health services for adolescents, there are significant resource challenges in training sufficient health professionals to meet this demand and providing the required infrastructure and supplies. While internet-based self-help therapies hold promise of lower costs (Stelmach et al. 2022), adequately meeting demand in resource constrained contexts is difficult. A companion article in this supplement discusses the options for funding scale up of services across the spectrum of adolescent well-being.

## Conclusions

Investment in mental health treatment is not only important from a human rights perspective to reduce disability, distress, self-harm, and suicide among adolescents, it also proves to be an effective investment supporting national economic development, especially in countries with a rapidly increasing adolescent population.

Ross et al. (2022) have set out four reasons for increasing investment in adolescents and their well-being, including mental health. They argue that investing in adolescents is a basic duty of governments, to address adolescent well-being as a human rights imperative. Governments and adults have a duty to support their well-being and to empower them to realize their rights of: good health and optimum nutrition; connectedness; positive values and contribution to society; safety and a supportive environment; learning, competence, education, skills, and employability; and agency and resilience.

Secondly, the adverse consequences of the climate emergency and the changing nature of work, with higher skill requirements and fewer entry level jobs, have increased the urgency of equipping this rapidly growing segment of the population with skills and other capabilities.

Thirdly, investing in adolescents and their well-being makes sound economic sense because many of the benefits of those investments are not only immediate, but also endure for the rest of their lives.

Finally, investing in adolescents and their well-being strengthens the human capital (accumulated knowledge, skills, and health) of a country, and hence the country’s potential for future development, and for ending extreme poverty and creating more inclusive societies.

The ROIs for Colombia and South Africa and those reported in the other ROI studies discussed earlier are sufficiently high to justify and convince policymakers in other countries to make similar investments on improving adolescent health, especially for the treatment of depression and anxiety among adolescents in LMICs. Although there is strong evidence for treatment and prevention programmes in HICs (Thomas et al. 2026), further research is required to establish the investment case for internet-based self-help therapies and other low-cost interventions in LMICs.

The findings of this study in two UMICs (Colombia and South Africa) suggest the following policy priorities for LMICs:

1. Prioritize adolescent mental health in national health strategies and budgets. Health ministries should integrate the treatment of common mental disorders (depression and anxiety) among adolescents explicitly within the national health and universal health coverage plans, allocating dedicated funding for scalable, evidence-based interventions.
2. Scale up cost-effective, evidence-based interventions for the treatment of depression and anxiety in adolescents. The investment case demonstrates strong returns of up to 15.4 BCR in Colombia and 13.9 in South Africa, when treating adolescent depression and anxiety. Governments should expand access to psychosocial interventions like CBT especially in schools and community settings.
3. Explore the feasibility, cost, and effectiveness of school-based and digital delivery platforms. Given the accessibility of schools and the increasing role of digital tools, both Ministries of Education and Health should collaborate to explore internet-based therapies.
4. Address the adolescent mental health workforce gap. Implementation is constrained by limited human resources even with strong ROI. Governments and partners should explore the feasibility and effectiveness of training non-specialist providers (school counsellors, community health workers) to deliver first-line mental healthcare especially in primary care and educational settings.
5. Build routine mental health data systems and monitoring frameworks. Data gaps remain a major barrier to planning and resource allocation. National health information systems should be strengthened to include age-disaggregated data on adolescent mental health and on treatment coverage.

## Data Availability

The data underlying this study were obtained from publicly available third-party sources. Details of the datasets and their access links are provided in the relevant references listed in the reference list.

## Reflexivity statement

The research originated from a global study into the urgent need for investment in adolescent well-being especially in LMICs. The authors include eight women and two men, all specialists in various areas of public health research and health policy across multiple levels and countries. Four authors are residents of, or primarily affiliated with institutions in, LMICs. The study has benefitted greatly from the considerable expertise in their work across varied geographies and covering public health practice, economic modelling and analysis, research, and policy making.
